# Human infection with *Candidatus Rickettsia* jingxinensis: First identification and clinical characteristics

**DOI:** 10.1371/journal.pntd.0013685

**Published:** 2025-11-05

**Authors:** Yuan Gao, Yu Wang, Lingyuhui Du, Xuesheng Liu, Yingwei Sun, Yi Zhang, Han Wu, Shujun Zhang, Zhiqian Wang, Lingling Mao, Baocheng Deng

**Affiliations:** 1 The second Department of Infectious Diseases, The First Hospital of China Medical University, Shenyang, Liaoning Province, China; 2 National Clinical Research Center for Laboratory Medicine, Department of Laboratory Medicine, The First Hospital of China Medical University, Shenyang, Liaoning Province, China; 3 China Medical University, Shenyang, Liaoning Province, China; 4 Liaoning Center for Disease Control and Prevention, Shenyang, Liaoning Province, China; 5 Department of Infectious Disease, Kuandian Country Hospital, Dandong, Liaoning Province, China; 6 School of Public Health, China Medical University, Shenyang, Liaoning Province, China; University of Kentucky College of Medicine, UNITED STATES OF AMERICA

## Abstract

**Background:**

New tick-borne pathogens are being discovered worldwide, and recognized tick-borne diseases are becoming increasingly diverse. Candidatus R. jingxinensis is endemic in Asia, but its potential to cause clinical infection in humans remains unclear. This study was designed to elucidate the prevalence and delineate the clinical profile of *Candidatus* Rickettsia jingxinensis infection in Liaoning Province, China.

**Methods:**

The subjects of this study were suspected cases of tick-borne infectious diseases admitted to the First Affiliated Hospital of China Medical University or reported to the Liaoning Provincial Center for Disease Control and Prevention in 2018–2022. Epidemiological and clinical data were collected. Tick-borne pathogens were detected with a microfluidic chip detection system, and specific gene fragments of the screened pathogens were amplified, sequenced, and compared. Evolutionary and phylogenetic trees were constructed and analyzed.

**Results:**

In total, 398 infected subjects from 14 cities were included in the study, and 255 tick-borne pathogens were detected. Among these, 11 subjects were found to be infected with *Candidatus* Rickettsia jingxinensis. This is the first time this strain has been shown to cause infection and illness in humans. The main clinical features of subjects infected with *Candidatus* R. jingxinensis included fever, fatigue, dizziness, headache, nausea, diarrhea, general pain or muscle and joint pain, reduced leukocytes and platelets, abnormal coagulation function and liver function.

**Conclusions:**

This study documents the first human infections with *Candidatus* R. jingxinensis, confirms its prevalence in Liaoning Province, and delineates the primary clinical manifestations of the disease.

## Author summary

No clinical cases of *Candidatus* R. jingxinensis infection had been reported prior to this study. During our screening of patients for common tick-borne pathogens known to be present in ticks from Liaoning Province, we identified eleven individuals infected with *Candidatus* Rickettsia jingxinensis—a species first discovered in 2016 in two pools of Haemaphysalis longicornis nymphs collected in Jingxin City, Jilin Province, China. This represents the first evidence that this strain can cause infection and disease in humans. The primary clinical manifestations observed in these patients included fever, fatigue, dizziness, headache, leukopenia, thrombocytopenia, abnormal coagulation function, and hypoalbuminemia. We believe our findings will assist clinicians in recognizing and managing infections caused by *Candidatus* R. jingxinensis.

## Introduction

Tick-borne infectious diseases (TBDs) are natural epidemic diseases predominantly caused by the bites of ticks carrying pathogens to human and animal hosts. Ticks carry more zoonotic pathogens than any other vector arthropods [1–4]. With the continuous expansion of human activities, the adaptation of ticks and tick-borne pathogens to climate and environmental changes, and the geographic expansion of ticks, TBDs pose an increasing threat to the health of their human and non-human hosts. New pathogenic tick-borne pathogens are being discovered worldwide, and recognized TBDs are becoming increasingly diverse. Since the 1950s, as many as 44 tick-borne pathogens have been detected in China, including viruses, rickettsias, spirochaetes, protozoa, etc. [5,6]. To 2018, 11,995 cases of severe fever with thrombocytopenia syndrome (SFTS) were reported, 2,786 individuals infected with *Spirochaeta*, 415 infected with *Anaplasma*, 215 infected with *Babesia*, 129 infected with spotted fever group *Rickettsia* (SFGR), 120 infected with *Francisella tularensis*, and 95 suffering Q fever in China [6,7]. SFGR infection, in particular, has been drawing increasing attention both in China and worldwide [8,9].

SFGR is highly endemic, and emerging species such as *Candidatus* Rickettsia xinyangensis have been identified as causative agents of clinical infection [10]. *Candidatus* R. jingxinensis was first detected in 2016 in two pools of Haemaphysalis longicornis nymphs in Jingxin City, Jilin Province, China, from which the pathogen derives its name [11]. In recent years, *Candidatus* R. jingxinensis has been reported in H. longicornis and other host animals, including cattle and sheep, across multiple Chinese provinces such as Shaanxi, Guizhou, Yunnan, Guangxi, Jiangsu, and Sichuan—in many of these regions as the dominant species [12–14]. It is noteworthy that the pathogen has also been detected in South Korea, near China’s Liaoning Province [15]. However, no previous studies had documented clinical human infection with *Candidatus* R. jingxinensis.

In this study, we provide the first confirmation of clinical *Candidatus* R. jingxinensis infection in Liaoning Province and summarize the clinical characteristics of its infection. These findings are expected to aid clinicians in the diagnosis and management of infections caused by this emerging pathogen.

## Methods

### Ethics statement

We adhering to ethical standards established by the 1964 Helsinki Declaration and its subsequent updates. To safeguard patient privacy, all data were anonymized and personal information was appropriately de-identified. The study received approval from the Ethics Committee of China Medical University in Shenyang China (Approval number: AF-SOP-07-1.1-01). Ensuring no adverse impact on subjects’ rights or welfare, informed consent was signed.

### Study design

**Fig 1:**
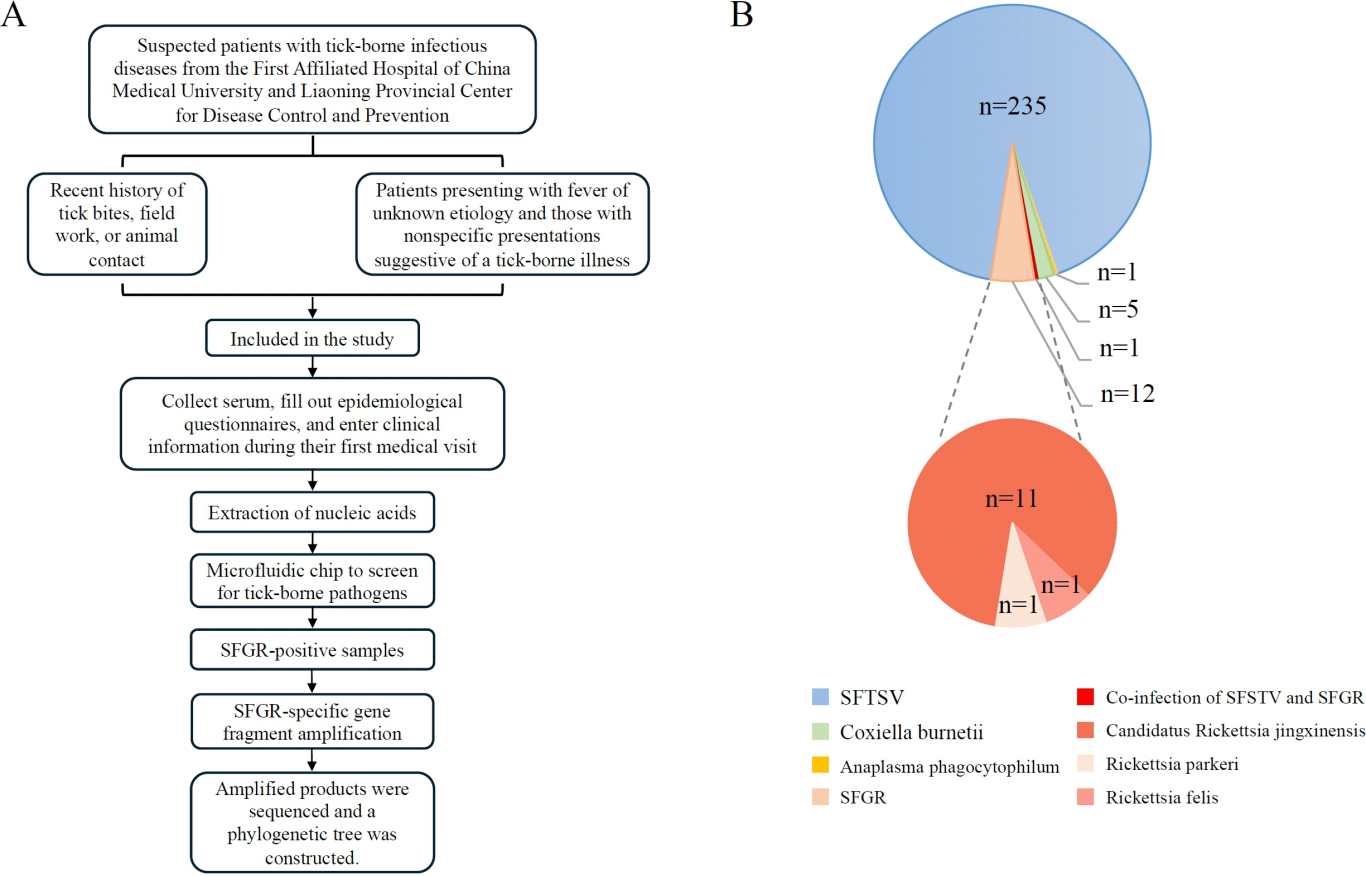
Study overview. **(A)** Flow chart of the study design. **(B)** Distribution of tick-borne pathogens. The top panel illustrates the overall composition of detected tick-borne pathogens, with case numbers and colors representing different species. The bottom panel details the species-level diversity within the SFGR, with case numbers and colors corresponding to different SFGR species. SFTSV: Severe fever with thrombocytopenia syndrome virus; SFGR: Spotted fever group rickettsia.

The subjects of the study were patients with suspected tick-borne infectious diseases treated at the First Affiliated Hospital of China Medical University in Shenyang China or reported to Liaoning Provincial Center for Disease Control during the five years from 2018 to 2022. After the consent of the patients was given, serum samples, epidemiological information, clinical manifestations, and test indicators of the patients were collected (Fig 1A).

Inclusion criteria: (1) Epidemiological history: a clear history of tick bite prior to symptom onset; a history of living or working in a tick-endemic area; or a history of contact with animals or diseased animals. (2) Fever (body temperature ≥ 37.5°C) in patients for whom the pathogen could not be identified with routine testing. (3) Other clinical manifestations: headache, fatigue, muscle or joint pain, nausea and vomiting, abdominal pain and diarrhea, body hemorrhage, petechiae, or other symptoms of nonspecific TBD.

Exclusion criteria: Lack of epidemiological information; short admission period; or no detailed clinical information or laboratory tests.

### Research methods

In this study, an epidemiological questionnaire was completed by patients with suspected TBD, which mainly collected general information (name, age, sex, occupation, current address) and epidemiological information (tick-bite history, field work history, tick activity in the residence, animal rearing conditions, etc.) on the patients. Clinical information (time of onset, signs and symptoms, laboratory test indicators, medication and course records, and prognosis) were also recorded.

Serum samples (3 ml) were collected from all subjects included in the study during their first medical visit, and the supernatant was extracted by centrifugation (Sorvall ST4 Plus; Thermo Fisher Scientific, Massachusetts, USA) at 3000 rpm for 10 min. The EZ1 Virus Mini Kit v2.0 (QIAGEN, Dusseldorf, Germany) was used to extract the nucleic acid. The nucleic acid concentrations of the samples were determined with a spectrophotometer (NanoDrop; Thermo Fisher Scientific).

### Microfluidic chip technology

A customized microfluidic chip pre-embedded with 10 conserved genome regions for eight tick-borne pathogens known to be carried by ticks in Liaoning Province was used, as described in previous research [16,17] (Table 1). We simultaneously screened for eight tick-borne pathogens with the Gene Expression Micro Fluidic Card (Thermo Fisher Scientific) and Real-time fluorescence quantitative PCR instrument (ABI7500, Thermo Fisher Scientific).

**Table 1:** The target and reagent ID of 10 genome conserved regions for 8 tick-borne pathogens.

Target gene	Reagent ID
Severe Fever with Thrombocytopenia Syndrome Bunyavirus	APH6E4P
Spotted fever group rickettsiae (OmpA)	APWC3H3
Spotted fever group rickettsiae (17-kDa)	APXGW3Z
Spotted fever group rickettsiae (gltA)	APT2FD9
Coxiella burnetii	AI89L3T
Anaplasma phagocytophilum	APZTGMF
Ehrlichia sp	APGZK3R
Babesia sp	APNKXEN/APPRRYK
Borrelia burgdorferi	APFVN6G
FT_23kda_ISFtu2	APEPX4A/APFVTN7

### Gene amplification and capillary electrophoresis

Nested PCR amplifier (EASTWIN, Beijing, China) was used to amplify fragments of the 17-kDa (Table 2) and ompB (Table 3) antigen genes from the SFGR-positive specimens, and the amplicons were analyzed with capillary electrophoresis (QIAxcel Advanced, QIAGEN). The primers used for gene amplification were from Sangon Biotech (Shanghai, China).

**Table 2:** The sequences of the 17-kDa primers.

Primer	Sequence (5’ -3’)	Amplified fragment length
17kd5	GCTTTACAAAATTCTAAAAACCATATA	434 bp
17kd3	TGTCTATCAATTCACAACTTGCC	
17kd1	GCTCTTGCAACTTCTATGTT	
17kd2	CATTGTTCGTCAGGTTGGCG	

**Table 3:** The sequences of the ompB primers.

Primer	Sequence (5’ -3’)	Amplified fragment length
rompB OF	GTAACCGGAAGTAATCGTTTCGTAA	384 bp
rompB OR	GCTTTATAACCAGCTAAACCACC	
rompB SFG IF	GTTTAATACGTGCTGCTAACCAA	
rompB SFG/TG IR	GGTTTGGCCCATATACCATAAG	

### Gene sequencing and analysis

The positive PCR products were subjected to first-generation sequencing by Tianyi Huiyuan Co., Ltd (Beijing, China). Phylogenetic analysis was conducted using PhyloSuite v1.2.3 [18]. Initially, homologous sequences for the 17-kDa and ompB genes were retrieved from the NCBI database via BLASTn. The obtained sequences were subsequently aligned using MAFFT v7.313 [19], implemented within PhyloSuite, and the alignments were concatenated into a single matrix using the built-in sequence concatenation function. The best-fit partitioning scheme and nucleotide substitution models were then determined under the corrected Akaike Information Criterion (AICc) using Model Finder, which employs a greedy search algorithm coupled with branch length linkage. Maximum likelihood (ML) phylogenetic trees were constructed with IQ-TREE, also integrated in PhyloSuite, applying the edge-linked partition model. Branch support was assessed with an ultrafast bootstrap approximation (10,000 replicates). Finally, the resulting phylogenetic tree was visualized and annotated using the online ITOL tool (https://itol.embl.de/).

## Results

### Screening for tick-borne pathogens

Three hundred ninety-eight patients with suspected tick-borne pathogen infections examined in 2018–2022 were included in this study. Ultimately, 255 patients infected with tick-borne pathogens were identified, including 236 patients infected with SFTSV, 13 patients infected with SFGR, five patients infected with *Coxiella burnetii*, and one patient infected with *Anaplasma phagocytophilum*. There was a co-infection of SFTSV with SFGR (Fig 1B).

Among the 13 cases of SFGR infection, two were infected with *R. parkeri* and *R. felis,* respectively. while the remaining 11 patients were infected with *Candidatus* R. jingxinensis.

### Gene sequencing and phylogenetic analysis of SFGR-positive samples

**Fig 2:**
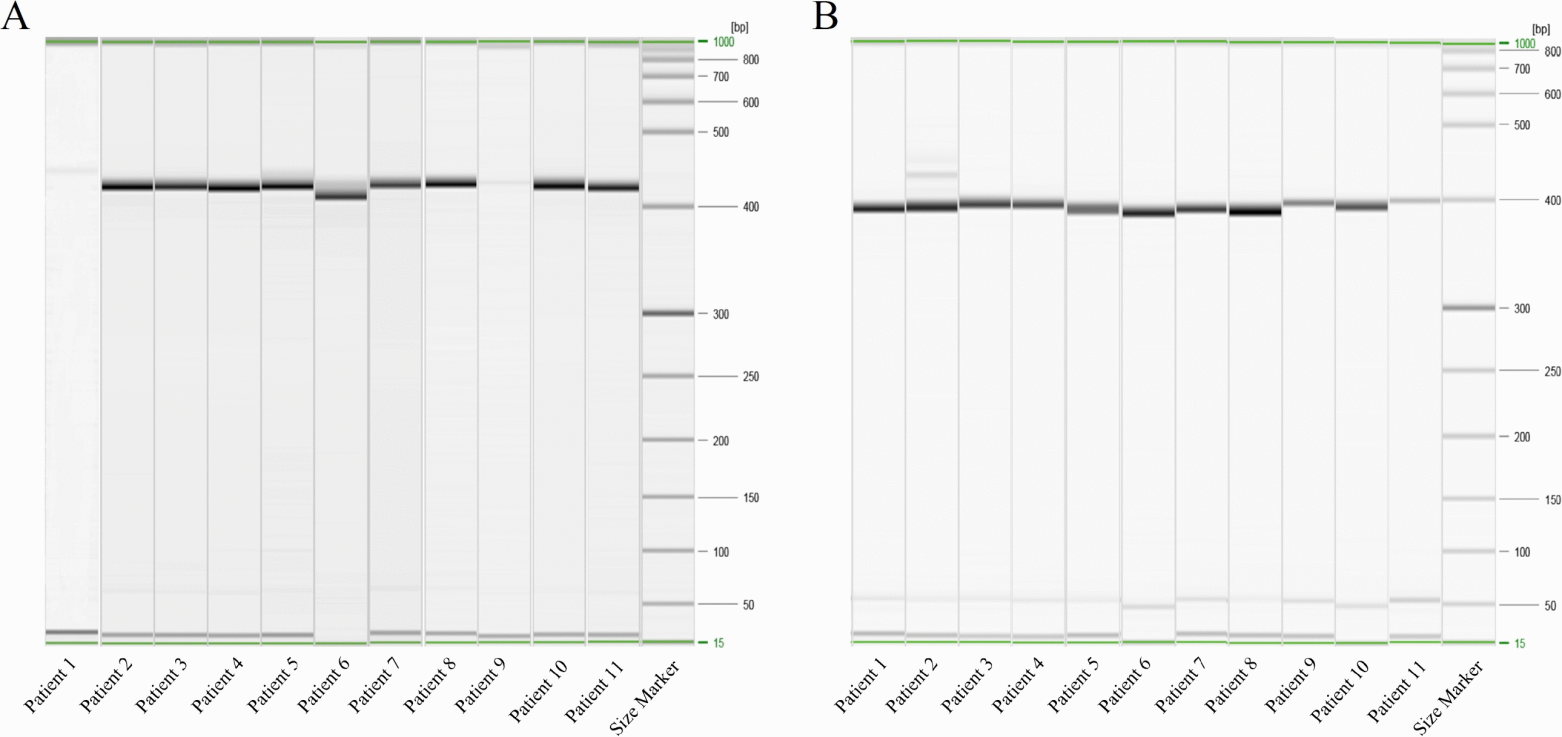
Nested PCR detection of spotted fever group rickettsia (SFGR) genes. Electropherogram shows the amplification of (A) the 17-kDa antigen gene and (B) the outer membrane protein B (ompB) gene fragments. Lanes 1-11 represent PCR products from different clinical samples. The rightmost lane contains a 1000-bp DNA ladder used as a molecular weight standard, with key band sizes (bp) annotated alongside. The green lines highlight the size range of the molecular weight marker, with the corresponding minimum and maximum values (in bp) indicated.

**Fig 3:**
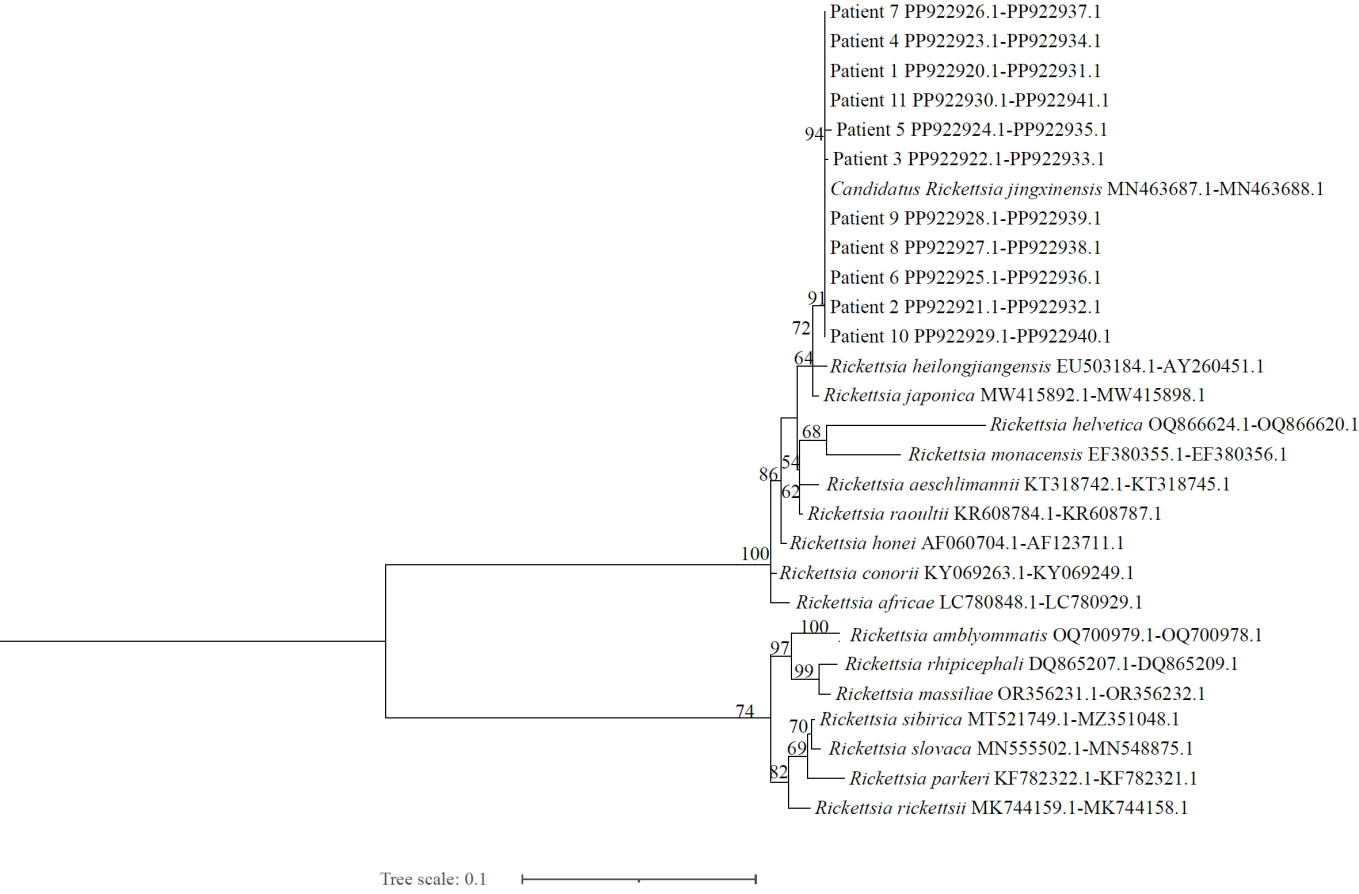
Phylogenetic analysis of spotted fever group rickettsia (SFGR) isolates. The tree was constructed from the concatenated alignment of the 17-kDa and ompB gene fragments. Bootstrap support values (10,000 replicates) are shown at the nodes. The horizontal branch lengths are drawn to scale, with the bar at the bottom representing 0.1 nucleotide substitutions per site.

Nested PCR and capillary electrophoresis were performed on the 11 SFGR-positive samples. The 434-bp PCR product amplified from the 17-kDa antigen gene (Fig 2A) and the 384-bp PCR product from the *ompB* gene (Fig 2B) were analyzed with DNA sequencing. Phylogenetic trees were constructed with the 99.11%–100% consistent sequences determined by BLAST comparison at NCBI, and other SFGR which were confirmed pathogenic around the world. On the phylogenetic trees based on the 17-kDa antigen and *ompB* genes (Fig 3), the 11 rickettsial strains were distributed in the SFGR clade, and were closely related to *Candidatus* R. jingxinensis (gene sequence number: MN463687.1 and MN463688.1). *Candidatus* R. jingxinensis detected in this study and the other pathogenic *Rickettsia* species reported in China (*R. heilongjiangiensis* and *R. japonica*) localized to the same large branch of SFGR.

### Clinical characteristics of Candidatus R. jingxinensis infection

One of the 11 patients with *Candidatus* R. jingxinensis infection had a history of tick bites, and two patients died (Tables 4 and 5). All patients had fever, with a peak temperature range from 37.7-40.0 °C. Other symptoms included fatigue (8/11 cases), dizziness and headache (6/11 cases), nausea (5/11 cases), diarrhea (5/11 cases), general pain or muscle and joint pain (5/11 cases), skin rash (4/11 cases), vomiting (4/11 cases), petechiae or nasal mucosal hemorrhage (3/11 cases), cough (2/11 cases), and abdominal pain (one case) (Table 4). On laboratory tests, eight patients showed reduced white blood cell (WBC) counts; nine patients showed reduced platelet counts; nine patients had abnormal coagulation function; alanine amino transferase (ALT) and apartate aminotransferase (AST) were elevated in five patients (> 2 × Upper Limit of Normal); total bilirubin (TBIL) was elevated in three patients; and creatinine was elevated in three patients (Table 5).

**Table 4:** Demographic characteristics, comorbidities, and clinical manifestations of patients with *Candidatus* R. jingxinensis infection.

	Patient1	Patient2	Patient3	Patient4	Patient5	Patient6	Patient7	Patient8	Patient9	Patient10	Patient11
Demographic characteristics											
Age	63	51	36	63	69	30	57	36	56	37	73
Sex	male	female	male	male	female	female	male	female	male	female	male
Occupation	woodworker	farmer	farmer	farmer	farmer	other	farmer	farmer	farmer	farmer	farmer
History of tick bites	×	×	×	×	×	√	×	×	×	×	×
History of contact with intermediate hosts	√	√	√	√	√	×	√	√	√	√	√
Comorbidity	×	×	×	×	×	×	×	×	×	×	×
Hypertention	×	√	×	×	×	×	×	×	√	×	×
Diabetes	×	√	×	×	×	×	×	×	√	×	×
Coronary heart disease	×	×	×	×	×	×	×	×	×	×	×
Chronic lung disease	×	×	×	×	×	×	×	×	×	×	×
Cerebrovascular disease	×	×	×	×	×	×	×	×	×	×	×
Renal insufficiency	×	×	×	×	×	×	×	×	×	×	×
Chronic liver disease	×	×	×	×	×	×	×	×	×	×	×
Malignant tumors	×	×	√	×	×	×	×	×	×	×	×
Other	×	×	×	×	×	×	×	×	×	×	psoriasis
Clinical features											
Fever peak (°C)	37.7	38	40	39.4	39.9	39.5	39	39	39.8	38	39
Dry cough	×	×	√	×	×	×	×	√	×	×	×
Sore throat	×	×	×	×	×	×	×	×	×	×	×
Dyspnea	×	√	×	×	×	√	×	×	×	×	×
Headache	×	×	×	×	×	√	×	√	×	×	√
Dizziness	√	×	√	√	×	×	×	×	×	×	√
Disturbance of consciousness	×	√	×	×	×	×	×	×	×	×	×
Anorexia	√	×	√	√	√	√	×	×	√	√	×
Nausea	×	×	×	√	√	√	√	×	×	×	√
Vomiting	√	×	×	×	√	√	×	×	×	×	√
Abdominal pain	×	×	×	√	√	√	×	×	√	×	√
Diarrhea	×	×	×	×	×	×	√	×	×	×	×
Constipation	×	×	×	×	×	×	×	×	×	×	×
Bloody stools	×	×	×	×	×	×	×	×	×	×	×
Muscle and joint pain	√	×	×	√	√	×	×	√	√	×	×
Fatigue	√	×	√	√	√	√	√	×	×	√	√
Rash	×	√	×	×	×	√	√	×	×	×	√
Petechia or ecchymosis	×	√	×	√	×	×	×	×	×	×	×
Bleeding	×	×	√	√	×	×	×	×	×	×	×
Other	×	×	×	occult blood in urine	conjunctival congestion		×	×	×	×	×

**Table 5:** Laboratory results, SFGR CT values, and clinical outcomes of patients with *Candidatus* R. jingxinensis infection.

	Patient1	Patient2	Patient3	Patient4	Patient5	Patient6	Patient7	Patient8	Patient9	Patient10	Patient11
Laboratory results											
WBC count (×10⁹/L)	1.79	5.46	0.66	1.56	4.59	1.29	0.5	2.6	5.2	1.5	3.1
PLT count (×10⁹/L)	20	5	5	2	52	67	54	203	112	55	29
Lymphocyte count (×10⁹/L)	0.45	0.05	0.42	0.28	0.47	0.39	0.2	0.71	1.19	0.68	0.57
% of lymphocyte in WBC	23.7	1.5	25.9	17.9	8	21.6	18.9	21.2	23.1	46.4	18.7
Neutrophil count (×10⁹/L)	0.76	2.19	0.05	1.15	3.52	0.81	0.2	1.2	3.43	0.66	1.98
% of neutrophil in WBC	34.4	89.4	4.8	73.8	71.3	62.8	14.6	46.8	66.5	45	42.2
Cr (μmol/L)	64	332	196	77	101	48	112	75	60.1	101	63.5
ALT (U/L)	321	261	983	144	18	205	151	25.2	43.2	44	34.9
AST (U/L)	95	42	990	187	31	200	277	19	25.3	67	50.2
ALP (U/L)	190	82	312	252	67	52	64	42.9	46	106	114.7
GGT (U/L)	252	93	463	341	22	31	34	21	26	63	167
TBIL (μmol/L)	15	7.5	171.4	134.3	20.4	13.3	22.9	15.49	10.61	28.2	11.23
DBIL (μmol/L)	6.3	4.5	155.3	116.5	5.5	4.7	12.7	6.48	5.99	22.2	6.4
ALB (g/L)	25.5	26.5	21.3	20.1	25.7	42.3	27.7	41.7	41	34.1	32.5
PT (s)	14.9	15.1	23.6	18.3	15.3	13.3	12.6	11.3	12.7	21.6	11.5
APTT (s)	37.5	26.5	86.3	70.9	50.8	62.5	57.6	39.5	38.7	69.5	33.5
TT (s)	22.7	19.4	69	27.2	17.2	23.9	24.3	15	14.5	27.9	15.4
CT value for SFGR	34.34	34.75	28.21	28.69	35.10	34.44	35.47	35.54	35.21	35.22	33.19
Clinical outcome	survival	survival	death	death	survival	survival	survival	survival	survival	survival	survival

WBC, White Blood Cell; PLT, Platelet; Cr, Creatinine; ALT, Alanine Aminotransferase; AST, Aspartate Aminotransferase; ALP, Alkaline Phosphatase; GGT, Gamma-Glutamyl Transferase; TBIL, Total Bilirubin; DBIL, Direct Bilirubin; ALB, Albumin; PT, Prothrombin Time; APTT, Activated Partial Thromboplastin Time; TT, Thrombin Time; SFGR, Spotted Fever Group Rickettsia.

Table 5 presents the laboratory indicators of these patients infected with *Candidatus* R. jingxinensis. The WBC and platelet counts were lower in the patients who died (S1 Fig), suggesting that low counts might be associated with poorer outcomes. TBIL, DBIL, alkaline phosphatase (ALP), and gamma-glutamyl transferase (GGT) levels were elevated in those who died (S1 Fig), suggesting that the impairment of hepatobiliary function might be linked to an increased risk of mortality and that these indicators are strong predictors of a poor prognosis. Albumin levels correlated positively with survival (S1 Fig), highlighting the positive impact of the nutritional status and liver function on patient survival. Coagulation times were generally prolonged in the patients who died, particularly the *activated partial thromboplastin time* (APTT) (S1 Fig), suggesting that coagulation disorders might be related to an increased risk of mortality.

## Discussion

The ticks analyzed in this study were collected from patients visiting the hospital, suggesting the possibility of human infection with *Candidatus* R. jingxinensis. Previously, *R. montanensis* was considered non-pathogenic, but it was later found to cause illness in humans [20]. Moreover, another *Candidatus* Rickettsia species was detected in humans in Xinyang City, Henan Province, in 2016, which was subsequently confirmed and named *Candidatus* R. xinyangensis [10]. The present study is the first to confirm *Candidatus* R. jingxinensis in human infections.

The combination of quantitative real-time PCR (qPCR) and serological testing is likely the optimal diagnostic approach. Although serum PCR has limited sensitivity for obligate intracellular bacteria, qPCR can detect early infection before antibodies appear [21,22]. In this study, all samples were collected during the early stage of illness, whereas serological confirmation generally requires at least two weeks after symptom onset; therefore, antibody testing was not performed. Rickettsial infection was primarily diagnosed using a customized microfluidic chip—a higher-throughput method compared to conventional PCR in this study. This molecular diagnostic approach has been reported in previous studies [23,24]. Further confirmation of *Candidatus* R. jingxinensis was achieved by PCR amplification and sequencing, followed by phylogenetic analysis; however, this approach has limitations compared to full genomic studies. Definitive species identification usually requires rickettsial isolation, which demands specialized laboratory equipment and appropriate safety measures, posing challenges in clinical practice [22,25,26]. We plan to perform genomic sequencing in future work to further characterize *Candidatus* R. jingxinensis [27].

Clinical manifestations of different SFGR are similar, including fever, headache, and prominent myalgia, often accompanied by rash or an eschar [27]. In this study, all 11 patients with *Candidatus* R. jingxinensis infections showed fever, six patients had headache, and rash was reported in only four patients. Fatigue, nausea, diarrhea, and general pain or muscle and joint pain were also common. Most patients had reduced WBC and platelet counts, and coagulation and liver function abnormalities. Although several key laboratory indicators (e.g., WBC, platelets, ALT, and AST,) differed significantly between the patients who survived and those who died, these finding cannot be confidently explained because the sample size was small. This suggests that although these indicators provide prognostic insights into the trend in survival, a diagnostic model and further validation with larger-scale studies are required for statistical rigor. Some studies suggest that rickettsiae are highly conserved and that excessive speciation may be unnecessary [25]; however, different strains exhibit considerable variation in virulence and prognosis, which influences clinical decision-making [28]. Such variation warrants differentiation among SFGR species [8]. Monitoring these clinically significant indicators and prevalence patterns may therefore help clinicians identify high-risk patients earlier and implement appropriate interventions.

The clinical manifestations of SFGR-infected patients are diverse and nonspecific, but we should be vigilant in the screening for and the diagnosis of *Rickettsia* infections. Unlike the symptomatic treatment of SFTS, doxycycline, a tetracycline antibiotic, has obvious efficacy in the treatment of rickettsial diseases. Although several studies have shown that a high bacteria load in the blood of rickettsial patients is uncommon [29,30], and the cycle threshold values of all the samples in this study were consistent with this view, there were still two deaths among our study subjects. Further investigation into the virulence mechanisms and drug susceptibility of *Candidatus* R. jingxinensis is warranted. Therefore, early diagnosis and timely treatment remain critical in SFGR infections.

This study has several limitations. First, the identification method requires further validation through pathogen isolation and sequencing. Second, the virulence characteristics of *Candidatus* R. jingxinensis relative to other rickettsiae need further comparative assessment. Third, the small sample size limits the generalizability of the clinical features and treatment experience described.

In conclusion, this study provides the first evidence of human infection with *Candidatus* R. jingxinensis in Liaoning Province, China. Our findings highlight the importance of clinical and epidemiological vigilance regarding this emerging pathogen.

## Supporting information

S1 Fig Comparison of clinical indicators between survival and death groups. Box plots show the distributions for each indicator.
(TIF)

S2 Fig Detection of tick-borne pathogens with the microfluidic chip technology in SFGR-infected patients. (TIF)
